# *Syn*-Propanethial S-Oxide as an Available Natural Building Block for the Preparation of Nitro-Functionalized, Sulfur-Containing Five-Membered Heterocycles: An MEDT Study

**DOI:** 10.3390/molecules29204892

**Published:** 2024-10-15

**Authors:** Mikołaj Sadowski, Ewa Dresler, Karolina Zawadzińska, Aneta Wróblewska, Radomir Jasiński

**Affiliations:** 1 Department of Organic Chemistry and Technology, Cracow University of Technology, Warszawska 24, 31-155 Krakow, Poland; mikolaj.sadowski@doktorant.pk.edu.pl; 2 Łukasiewicz Research Network—Institute of Heavy Organic Synthesis “Blachownia”, Energetyków 9, 47-225 Kędzierzyn-Koźle, Poland; ewa.dresler@icso.lukasiewicz.gov.pl; 3 Radom Scientific Society, Rynek 15, 26-600 Radom, Poland; karolina.zawadzinska@doktorant.pk.edu.pl; 4 Department of Organic Chemistry, University of Lodz, Tamka 12, 91-403 Łódź, Poland; aneta.wroblewska@chemia.uni.lodz.pl

**Keywords:** [3 + 2] cycloaddition, oxathiolanes, propanethial S-oxide, nitroalkenes, MEDT, ELF, docking affinity, target proteins

## Abstract

The regio- and stereoselectivity and the molecular mechanisms of the [3 + 2] cycloaddition reactions between *Syn*-propanethial S-oxide and selected conjugated nitroalkenes were explored theoretically in the framework of the Molecular Electron Density Theory. It was found that cycloadditions with the participation of nitroethene as well as its methyl- and chloro-substituted analogs can be realized via a single-step mechanism. On the other hand, [3 + 2] cycloaddition reactions between *Syn*-propanethial S-oxide and 1,1-dinitroethene can proceed according to a stepwise mechanism with a zwitterionic intermediate. Finally, we evaluated the affinity of model reaction products for several target proteins: cytochrome P450 14α-sterol demethylase CYP51 (RSCB Database PDB ID: 1EA1), metalloproteinase gelatinase B (MMP-9; PDB ID: 4XCT), and the inhibitors of cyclooxygenase COX-1 (PDB:3KK6) and COX-2 (PDB:5KIR).

## 1. Introduction

**Scheme 1:**
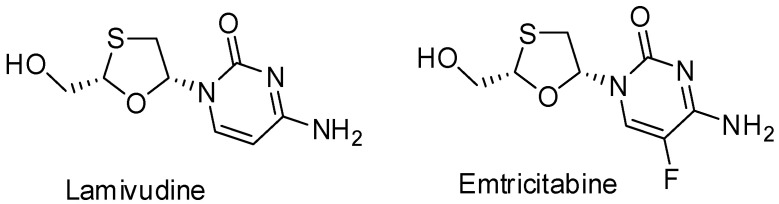
Examples of bioactive 1,3-oxathiolane derivatives.

Five-membered heterocycles, containing sulfur, play an important role in modern pharmaceutical and medical applications [1,2,3,4,5,6,7,8]. In particular, compounds of this type exhibit (*inter alia*) anticancer, antidiabetic, antimicrobial, antihypertensive, antiviral, and anti-inflammatory activities [9,10,11,12,13]. Within this group of heterocyclic compounds, especially interesting are oxathiolane molecular segments (Scheme 1). In the literature, many examples of the synthesis, transformations, and applications of 1,3-oxathiolane derivatives exist [14,15,16,17]. In contrast, far less is known about 1,2-oxathiolanes.

**Scheme 2:**
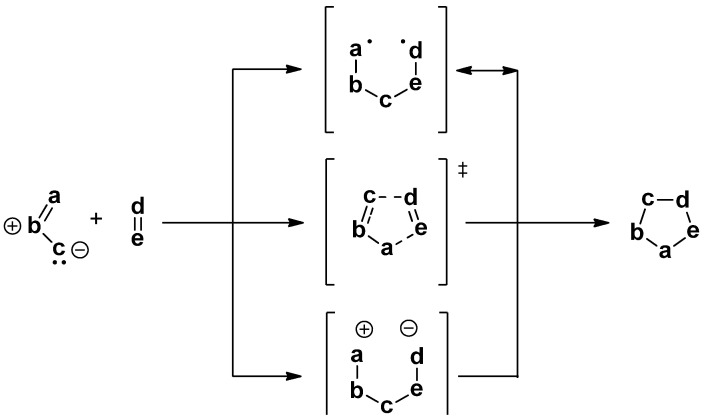
Single-step and stepwise mechanisms of [3 + 2] cycloaddition reactions.

The most universal protocol for the preparation of five-membered heterocycles consists of [3 + 2] (Scheme 2) cycloaddition (32CA) reactions [18,19,20,21]. As components for such transformations, different types of three-atom components (TACs) [22,23] including heteroatoms such as nitrogen, oxygen, and sulfur can be used. A popular methodology for the preparation of heterocyclic skeletons containing a single sulfur atom consists of 32CAs with the participation of thiocarbonyl ylides [24,25,26,27,28]. On the other hand, the preparation of 1,2-oxathiolanes is possible using unsaturated S-oxides. An interesting example of this type of TAC is propanethial S-oxide (**1**), which is a natural substance that is available from onion plants [29]. S-oxide (**1**) is a product of the enzyme-catalyzed rearrangement of 1-propenesulfenic acid [30]. The molecular structure and the *syn* conformation of propanethial S-oxide were determined by W. Niegisch and W. H. Stahl as well as by E. Block and co-workers. Only 5% of natural propanethial S-oxide exhibits the anti (E) configuration [31,32]. In the modern TAC classification, oxide **1** should be considered a bent-type cycloaddition component [33,34].

Within this work, we analyzed the possibility of the synthesis of new, nitro-functionalized analogs of 1,2-oxathiolane. As model 2π components in 32CA reactions with propanethial S-oxide (**1**), we selected nitroethene (**2a**) and its 1-substituted analogs (R = Me (**2b**), R = Cl (**2c**), and R = NO_2_ (**2d**)). Although 2-nitroprop-1-ene (**2b**) is known and has been described in relation to different types of chemical transformations, its properties as a component in 32CA processes is rather poorly known [35,36,37]. Additionally, nitroalkenes **2c**,**d** are not commonly mentioned in the literature [28,38,39,40,41]. It should be underlined that the application of conjugated nitroalkenes in 32CA processes creates the possibility of the preparation of heterocyclic systems characterized by additional bioactive stimulation by the presence of the nitro group [42,43,44]. It should be underlined at this point that the mechanism of the considered 32CA reactions cannot be assumed a priori. At present, two general groups of cycloaddition mechanisms (Scheme 1) are known, as defined by the nature of the intermolecular interactions between addends: polar mechanisms and non-polar mechanisms [45]. Within the first group, a single-step synchronous mechanism should be considered, but so should a single step non-synchronous mechanism and a stepwise mechanism with a zwitterionic intermediate [46,47]. On the other hand, non-polar mechanisms can also be realized via different types of schemes: a non-polar single-step synchronous mechanism, a single-step asynchronous mechanism, or a stepwise mechanism with a biradical intermediate [48].

Due to the issues mentioned above, we decided to proceed with a comprehensive exploration of the [3 + 2] cycloaddition reactions between *Syn*-propanethial S-oxide and selected conjugated nitroalkenes (CNA), including (i) an analysis of the global and local interactions between addends; (ii) an exploration of the kinetic and thermodynamic aspects of the model transformations; (iii) a full description of the energy profiles, including key parameters of critical structures; (iv) a prediction of the regio- and stereoselectivity; and (v) an interpretation of the cycloaddition mechanism. We hoped that implementing such a course of research would allow us to formulate general conclusions.

## 2. Results

**Scheme 3:**
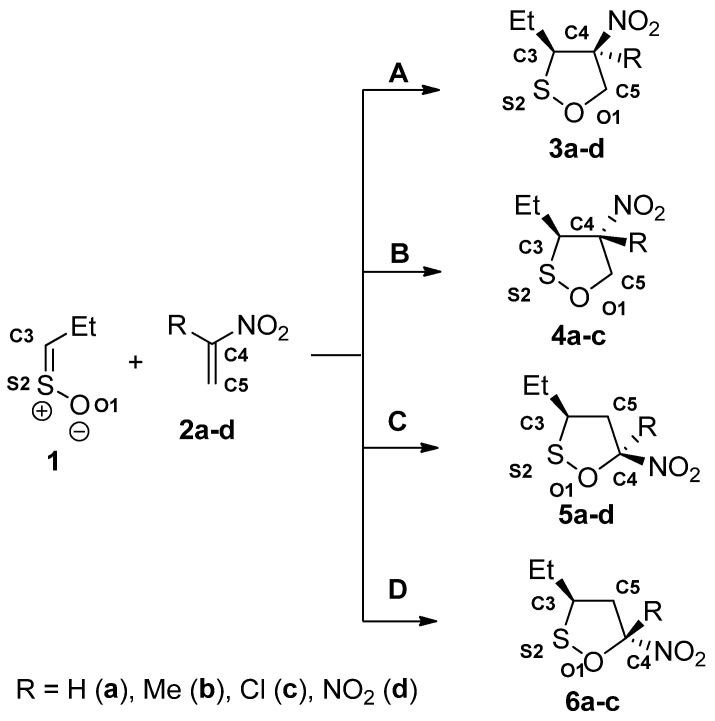
Theoretically possible regio- and stereoisomeric paths of the [3 + 2] cycloaddition between *Syn*-propanethial S-oxide (**1**) and 1-R-1-nitroethenes (**2a**–**d**).

The reaction mainly analyzed in our work is known as 32CAs and may theoretically lead to four regio- and stereoisomeric cycloadducts (Scheme 3). In the first part of our research, we decided to shed light on the nature of the interactions between addend molecules according to the reaction course.

Many electronic structures of various types of TACs have been analyzed within the Molecular Electron Density Theory [49], but S-oxides, even those similar to **1,** have not been described before. First of all, we decided to describe the electronic structure of **1**. Computational calculations for the compound were performed by Gaussian 16 revision C.01 [50] with B3LYP/6-31G(d) in the gas phase. The Parr function values were calculated with UB3LYP/6-31G(d) to fit into the unique electrophilicity/nucleophilicity scale proposed by Domingo et al. [51]. The level of theory was chosen according to Domingo’s recommendations, because this approach offers the possibility of a comparison with the universal reactivity scale [51]. The ELF values were calculated using the TopMod 09 software [52], and the ELF isosurface was rendered using the ParaView 5.9.1 software.

**Scheme 4:**
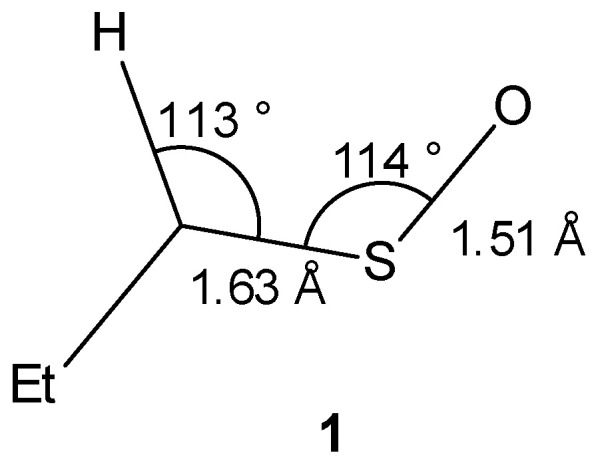
Geometry of the CSO group of oxide **1** calculated at the ground state.

The C=S-O moiety’s geometry is presented in Scheme 4. It is noticeable that the hydrogen atom connected to the sp^2^ carbon atom of the moiety is iso-planar relative to the C=S-O fragment. The C-S bond is 1.63 Å (elongated compared to circa 1.55 Å in CS2 [53]), and the S-O bond is 1.51 Å (slightly elongated compared to 1.43 Å in SO_2_ [54]).

**Scheme 5:**
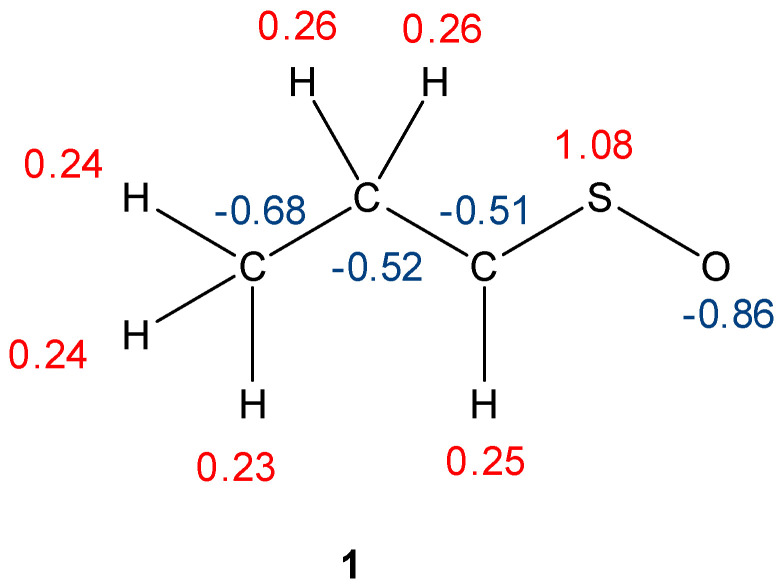
Natural charges of atoms (in electrons) in the S-oxide Xet, as computed at the ground state in the gaseous phase (B3LYP/6-31G(d)); charges > 0.2 e are given in red, and charges < −0.2 are given in blue.

Next, the natural population analysis for the compound was conducted. Computed values of natural charges are given near corresponding atoms in Scheme 5.

**Figure 1:**
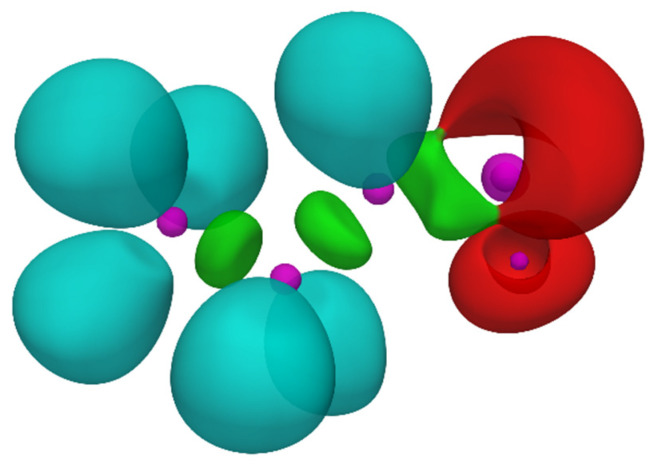
Topology of molecule **1** ELF, as rendered at an isovalue of 0.8. Core basins are given in magenta, protonated basins in cyan, disynaptic basins in green, and monosynaptic basins in red.

**Figure 2:**
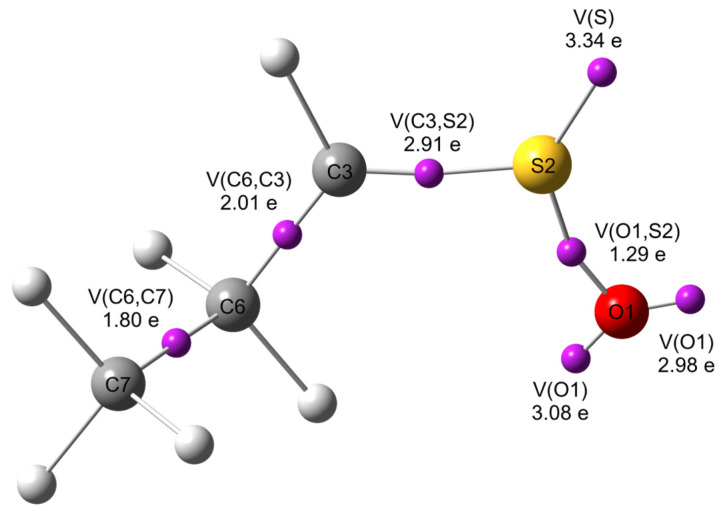
Positions and populations of ELF attractors of unprotonated basins in molecule **1**.

ELF topological and populational analysis was then conducted. A rendering of the molecule’s (**1**) ELF topology is shown in Figure 1. Populations of basins significant to the CSO group structure are given in electron as well as attractor positions and are shown in Figure 2.

In the ELF structure, a disynaptic basin with a single attractor between the carbon and sulfur atoms can be observed. V (C3, S2) has a population of 2.91 e, corresponding to a slightly underpopulated bond of 1.5 order. A monosynaptic valence basin V (S2) with a population of 3.34 e corresponds to an overpopulated lone electron pair of the sulfur atom. Between the oxygen atom and the sulfur atom, a disynaptic basin V (S2, O1) can be observed, with a population of only 1.29 e, which can be translated to a bond order of only 0.6. At the oxygen atom (at an isovalue of 0.8), a reducible [55] domain composed of two V (O1) valence basins is observed. The domain has a population of 6.06 e, which corresponds to three lone pairs of the oxygen atom.

Taking into consideration the population of the basin V (S2, O1) corresponding to the S2-O1 bond, the populations of the valence basins of the sulfur and the oxygen atoms, and the natural charges presented on the S2 and O1 atoms, it can be assumed that the S-O bond is of a donor–acceptor type. The proposed structure and the geometry of the molecule allows it to be classified as a TAC of an allyl type [18].

**Scheme 6:**
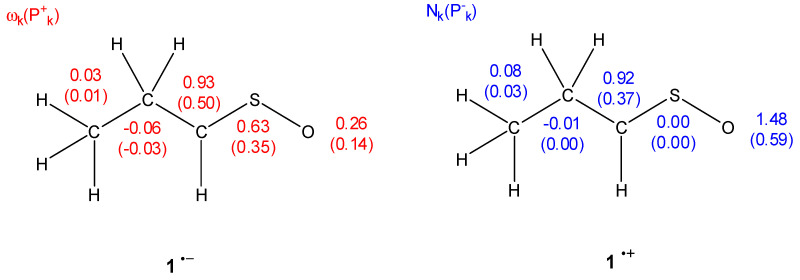
Parr function values for radical anion and radical cation of molecule **1**.

Considering molecule **1** as a TAC in the 32CA reaction, we analyzed the distribution of the local reactivities of this molecule. It was found that the global nucleophilicity of compound **1** was equal to 2.5 eV, while the global electrophilicity was 1.85 eV. These values categorize the compound as a strong electrophile and a moderate nucleophile according to the universal electrophilicity scale proposed by Domingo et al. [51]. Values of both the electrophilic (P*_k_*^+^) and nucleophilic (P*_k_*^−^) Parr functions as well as the local electrophilicity and nucleophilicity are shown in Scheme 6.

The most nucleophilic center of molecule **1** is located at the oxygen atom with N_O_ = 1.48 eV, while the most electrophilic center of oxide **1** is the C3 atom, with ω_C3_ = 0.92 eV.

Analogously, the electronic properties of conjugated nitroalkenes **2a**–**d** were described previously in detail [56,57,58]. The global electrophilicities of this group of compounds are in the range 2.43–4.52 eV. Within all the molecules, the most electrophilic center is located on the β-carbon atom of the nitrovinyl moiety. So, in the reaction with oxide **1**, the considered CNAs should be considered as electrophilic components, whereas oxide **1** should be considered as a nucleophilic agent. In these types of reactions, according to the Domingo rule [49,51,59], the regioselectivity should be determined by the interactions between the most electrophilic center of one component and the most nucleophilic center of the second one. In the considered processes, these interactions should be observed between the oxygen atom of molecule **1** and the β-carbon atom of the CNAs. Moreover, these types of interactions favor the formation of 4-nitro-substituted adducts.

**Figure 3:**
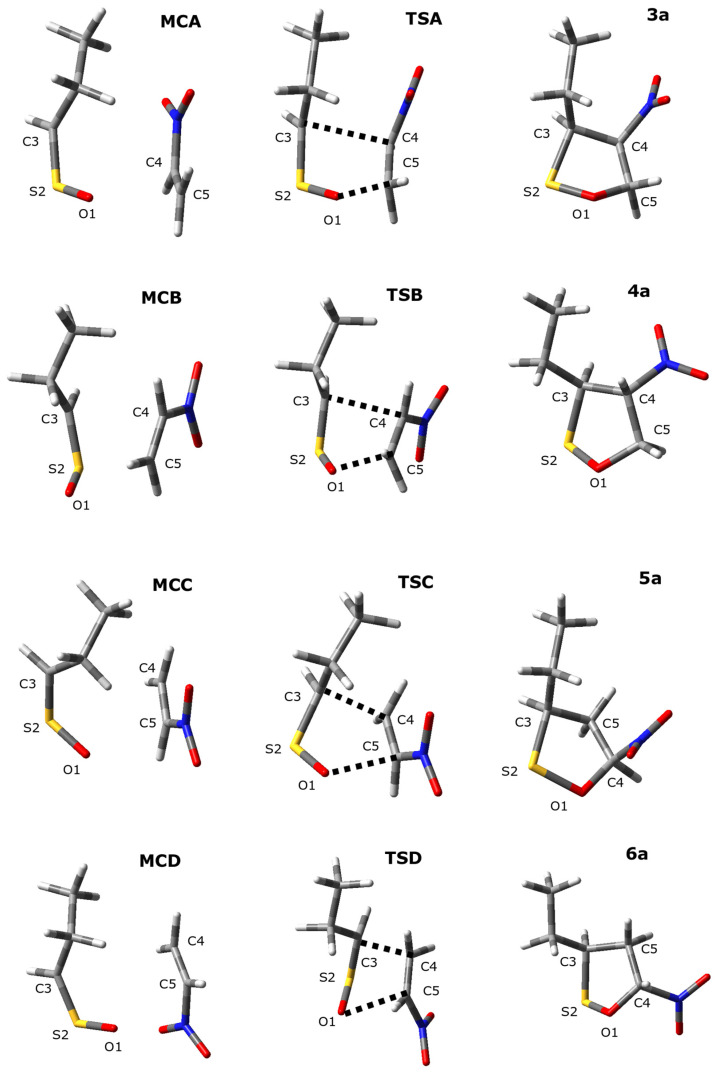
Views of critical structures for the [3 + 2] cycloaddition between *Syn*-propanethial S-oxide (**1**) and nitroethene **2a** in toluene solution according to wB97XD/6-31G(d) (PCM) calculations.

In the next step of our research, we explored the theoretically possible channels of 32CA. We started this analysis from the model reaction between oxide **1** and the parent nitroethene **2a**. It was found that the energy profiles for all the considered paths of **1** + **2a** cycloaddition in toluene solution were qualitatively similar. In particular, all the cases showed two critical points (a pre-reaction molecular complex, **MC,** and a transition state, **TS**), which were detected between the area of each individual reagent (from the first side) and the area of the respective product (from the second side). The conversion of the reaction system, at the first stage, led to the formation of **MC** complexes. The **1** + **2a→MC** transformation was barrierless. During this process, the enthalpy of the reaction was reduced by a few kcal/mol (Table 1). At the same time, the entropy of the reaction always substantially decreased. As a consequence, the Gibbs free energy of the formation of **MCs** demonstrated a positive value. This fact excludes the possibility of the existence of these **MCs** as thermodynamically stable and isolable intermediates. Analyzing both **MCs,** it could be said that the substructures adopted a mutual orientation, which determined their further transformation into the respective transition states. Ultimately, the detected structures should be considered as “orientation complexes”. Next, important information enabled the analysis of key interatomic distances. It was found that the O1-S2 and S2-C3 bonds as well as the C4-C5 bonds were almost identical, as was the case with the individual reagents (Table 2, Figure 3). On the other hand, the distances C3-C4 and C5-O1 were beyond the range of the typical respective bonds in transition states [60,61,62,63]. Finally, at this stage, any transfer of the electron density between substructures was not observed (GEDT = 0.00 e). Thus, the described structures were not charge-transfer complexes. It should be underlined that similar types of intermediates were recently detected in the profiles of different types of cycloaddition processes [64,65,66,67].

The further conversion of the **MC** intermediates led to the respective transition states (**TSA** for path **A**, **TSB** for path **B**, **TSC** for path **C**, and **TCD** for path **D**, respectively). This process required an increase in the enthalpy of the reaction of about 17–20 kcal/mol, depending on the reaction path. At the same time, the entropy of the reaction was reduced, which, in turn, caused an increase in the Gibbs free energies. From a kinetic point of view, the preference of the theoretically possible reaction paths can be presented in the order of **B** > **D** > **A** > **C**. Within the localized **TSs**, all the key interatomic distances adopted values typical for new single bonds in transition states [60,61,62,63]. It should be underlined that the distance near the β-carbon atom located in the nitrovinyl moiety was always substantially shorter than the second one. In turn, this confirmed the importance of the local nucleophilic/electrophilic interactions determined above. Similarly to the process of the formation of new single bonds, the electron density was transferred from the S-oxide substructure to the nitroalkene substructure (GEDT = 0.20–0.45 e). Thus, in light of Domingo’s terminology [68], the described cycloaddition reactions should be treated as Forward Electron Density Flux (FEDF) processes. For all the optimized transition structures, the full IRC trajectories were calculated. It was found that the intrinsic reaction vectors connected the **TSs** with the energetic valleys of the respective **MCs** and products. At this point, all attempts at the localization of the hypothetical zwitterionic intermediates were not successful.

The presence of a more polar solvent in the reaction environment resulted in only a very small effect on the kinetic aspects of the cycloaddition process as well as on the nature of the key structures (Table 1 and Table 2). In particular, considering the mentioned conditions, the activation barriers were slightly lower than in the toluene solution. However, the order of kinetic preference of the possible cycloaddition paths was always identical. Next, the asynchronicity of the transition states was slightly higher in comparison with the analogous structures optimized in toluene. Yet, these changes were not sufficient for the enforcing of stepwise cycloaddition mechanisms.

**Figure 4:**
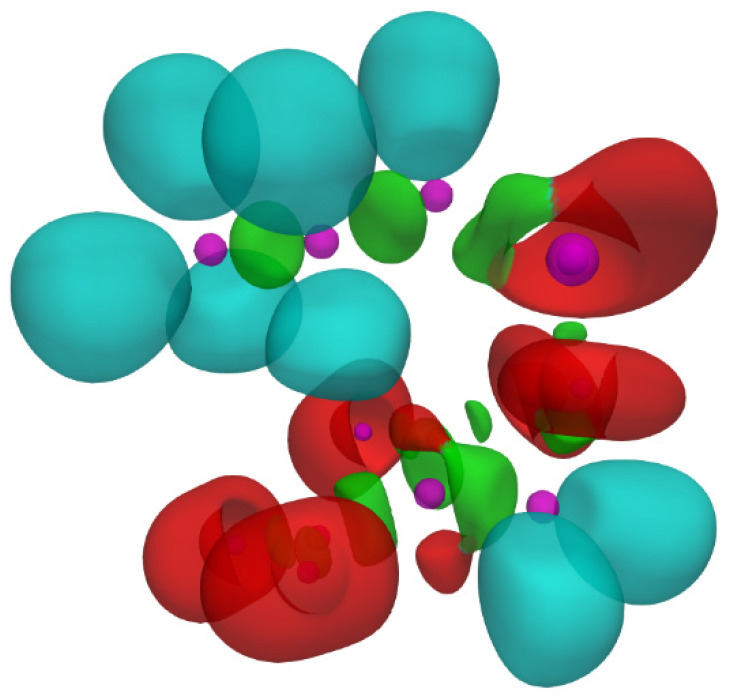
Visualization of ELF basins of system **IA** of reaction **1** + **2d** at an ELF isovalue of 0.8, as computed by wB97XD/6-31G(d) in toluene (PCM). Core basins are given in magenta, protonated basins in cyan, disynaptic basins in green, and monosynaptic basins in red.

**Figure 5:**
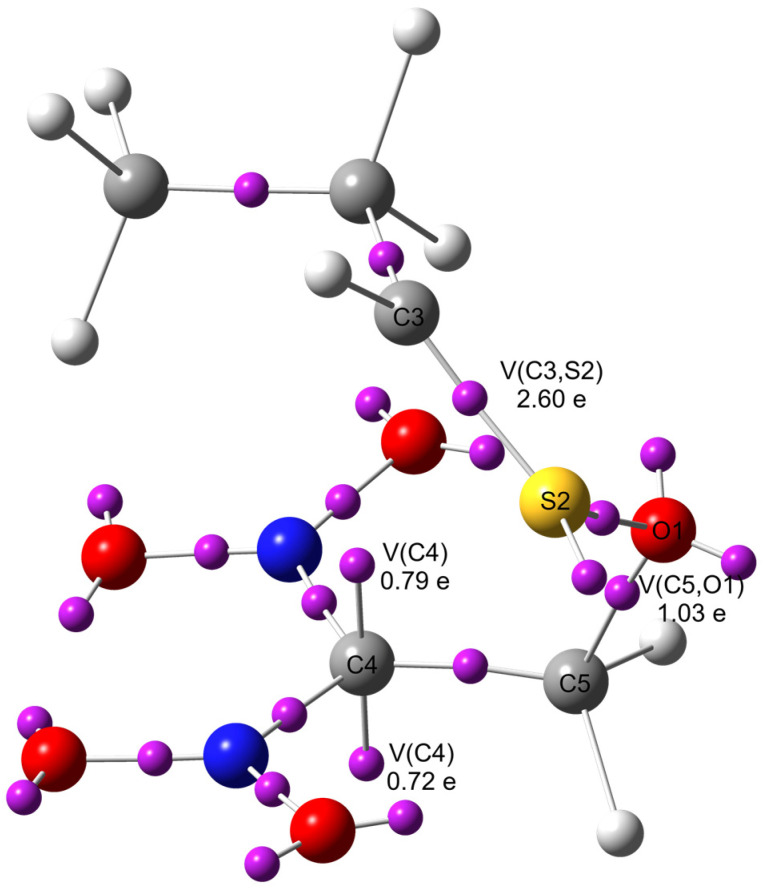
Populations of ELF attractors in intermediate **IA** of reaction **1** + **2d** and populations of significant basins, as computed by wB97XD/6-31G(d)) in toluene (PCM).

**Figure 6:**
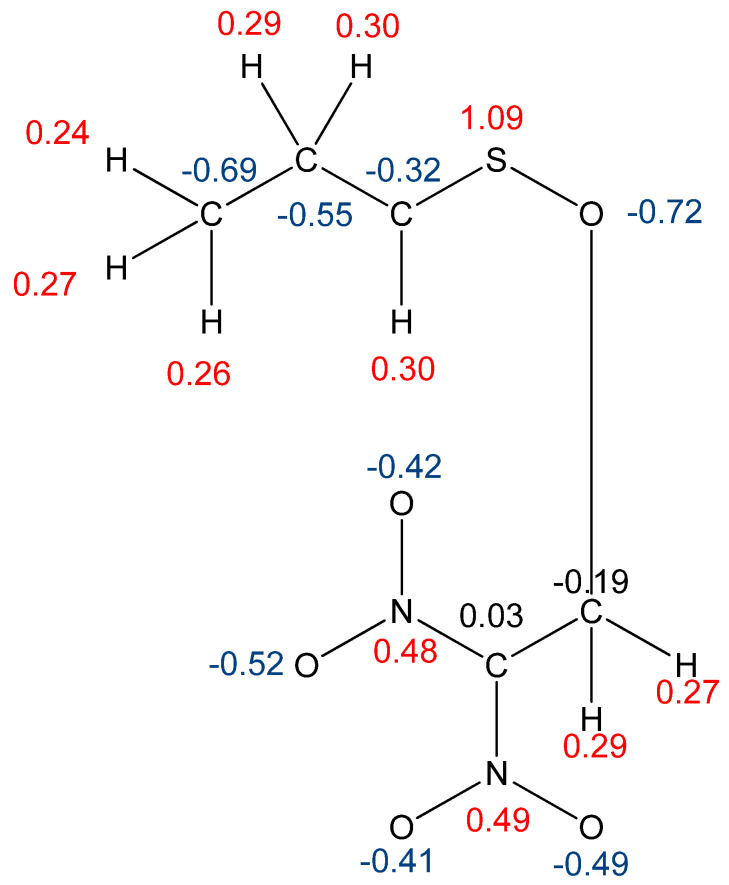
The natural charges of the atoms in intermediate **IA** of reaction **1** + **2d** (WB97XD/6-31G(d) in toluene (PCM)). Charges greater than 0.2 are given in red, those less than −0.2 are given in blue, and those between or equal to −0.2 and 0.2 are given in black.

To summarize, in our consideration of the field of the theoretical description of 32CA, the influence of the substituent nature on the reaction course and mechanism were evaluated. In the first step of this analysis, we explored the processes of 32CA with the participation of methyl- and chloro-substituted analogs of nitroethene (**2b** and **2c**, respectively). The nature of the energy profiles for these cycloadditions were similar to the case of **1** + **2a**. However, their description should be slightly different. From a kinetic point of view, the preference of the theoretically possible reaction paths can be presented in the orders of **D** > **B** > **C** > **A** and **D** > **B** > **A** > **C** for 32CAs **1** + **2b** and **1** + **2c**, respectively. On the other hand, asynchronicity of the respective **TSs** was more likely to be observed in the case of the **1** + **2a** reaction. It is interesting that the presence of the second nitro group in the 1-position of the nitrovinyl moiety changed the molecular mechanism drastically in the kinetically favored path A, which led to the 4,4-dinitrosubstituted adduct. In particular, between the energetic valleys of the reactants and the reaction product, we detected four critical points connected with the existence of the pre-reaction complex **MCA**, as well as two transition states (**TS1A** and **TS2A**) and an intermediate **IA**. The **MCA** complex was very similar to the complexes observed in the reaction between oxide **1** and the parent nitroethene **1a**. However, its conversion along the reaction coordinates led to the formation of only one new single bond. This was the O1-C5 bond (Figure 4). The formation of the mentioned single bond was advanced within the **TS1A** transition state. The further conversion of the reaction system led to the area of the acyclic intermediate **IA**. Its nature was explored in detail based on ELF analysis. The ELF of intermediate **IA** (Figure 5 and Figure 6) and the natural charges (Figure 6) of the system’s atoms were calculated by the methods described above for propanethial S-oxide.

No notable changes were observed in the part of structure **IA** corresponding toS-oxide **1** in comparison to the structure of the single S-oxide. The population of the C3-S2 bond decreased slightly, and a new bonding volume V (C5, O1) with a population of 1.03 e appeared, which translated to a bond order of 0.5. The two monosynaptic basins V (C4) located at the carbon atom adjacent to the nitro groups are very important. One basin has a population of 0.72 e, and the other has a population of 0.79 e.

The charges of the atoms in the part of the intermediate derived from S-oxide **1** did not change qualitatively; only slight changes are observed. What is significant is that the charge of the C4 carbon atom was equal to 0.03 e, effectively rendering the molecule slightly positively charged, considering the summarized population (1.51 e), the positions of the attractors, and the topological analysis of the ELF function near the C4 atom of intermediate **IA**. Despite only slightly positive charge on C4 carbon atom, we conclude that **IA** could be consider as the zwitterionic type, but only slightly polarized. Similar type of intermediates were previously observed on the paths of other 32CAs [45,69,70,71].

**Figure 7:**
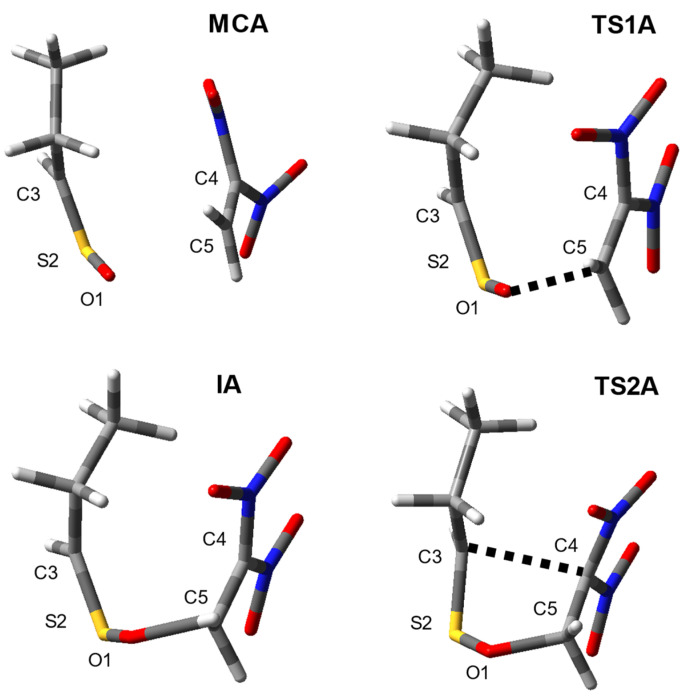
Views of critical structures for path A of the [3 + 2] cycloaddition between *Syn*-propanethial S-oxide (**1**) and 1,1-dinitroethene **2d** in toluene solution, according to wB97XD/6-31G(d) (PCM) calculations.

The further conversion of intermediate **IA** was connected to the formation of one new single C-C bond, i.e., the C3-C4 bond. The mentioned process was realized via transition state **TS2A**. The IRC calculations well connected **TS2A** directly with intermediate **IA** and product **5c**. This confirmed the before-proposed stepwise mechanism of the [3 + 2] cycloaddition reaction (Figure 7).

In the final part of our research, we decided to shed light on the potential bioactivity of the cycloadducts formed along the title reactions in the course of molecular docking. We evaluated the affinity of the tested compounds to several target proteins: cytochrome P450 14α-sterol demethylase CYP51 (RSCB Database PDB ID: 1EA1); metalloproteinase gelatinase B (MMP-9; PDB ID: 4XCT), and the inhibitors of cyclooxygenase COX-1(PDB:3KK6) and COX-2 (PDB:5KIR). First of all, we tested the affinity of 1,2-oxathiolanes to CYP51 (RSCB Database PDB ID: 1EA1) in order to estimate if the tested compounds could be used as antifungal agents. The determined affinities are shown in Table 3.

**Figure 8:**
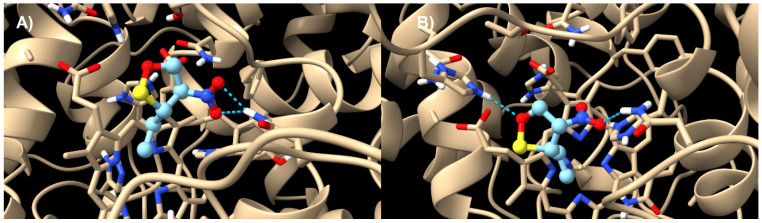
Visualization of hydrogen bond formation between ligand and protein for (**A**) **4a**, (**B**) **3a**, and COX-2 (PDB:5kir).

**Figure 9:**
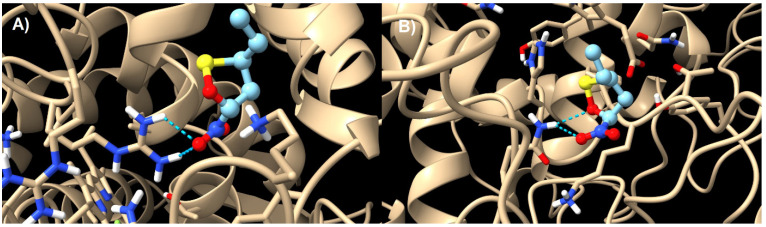
Visualization of hydrogen bond formation between ligand and protein for (**A**) **6a**, (**B**) **5a**, and CYP51 (PDB:1ea1).

The overall affinities of 1,2-oxathiolanes to CYP51 were in the range of from −3.35 to −3.79 kcal/mol. The affinity to this protein could be considered as rather medium compared to the same conditions that we used in the testing of isoxazolines and isoxazolidines, whose affinities were around−9 kcal/mol [42]. On the other hand, Pandey et al. [72] evaluated the docking scores for novel spirothiazolidinones to CYP51, and their scores were rather low, equal to −4.46 kcal/mol, leading to the conclusion that the introduction of sulfur into the heterocyclic chain could cause a lower binding energy to this protein. Next, we analyzed gelatinase B, which has been found to greatly induce the angiogenesis of cancer cells, in order to study its anticancer properties [42,73]. The best ΔG value was demonstrated by **3a,** and the worst was demonstrated by **6a**. This indicates that the cis position of the substituent could cause a worse binding with the pocket of the protein. This was noticed as a sort of a trend in all of the analyzed series. Moreover, the best affinity was demonstrated, in all cases, by **3a** and **5a**, and in this series of testing, it can be concluded that the trans position of the substituents improved the binding energy. The energies obtained due to the docking to COX-1 and COX-2 also showed rather low affinities in the range of from −4.48 to −5.48 kcal/mol in comparison to the well-known anti-inflammatory agent ketoprofen, which is in the range of −8.70 kcal/mol [74]. What is more is that the analysis of this series has also shown also that these compounds can create many hydrogen bonds with all of the mentioned proteins, mainly due to the presence of the nitro group and also the nitrogen atom in the heterocyclic chain (Figure 8 and Figure 9). This forms the basis for the confirmation of our previous statement, in that the presence of the nitro group can increase the great bio-active potential of these compounds [4,42].

**Figure 10:**
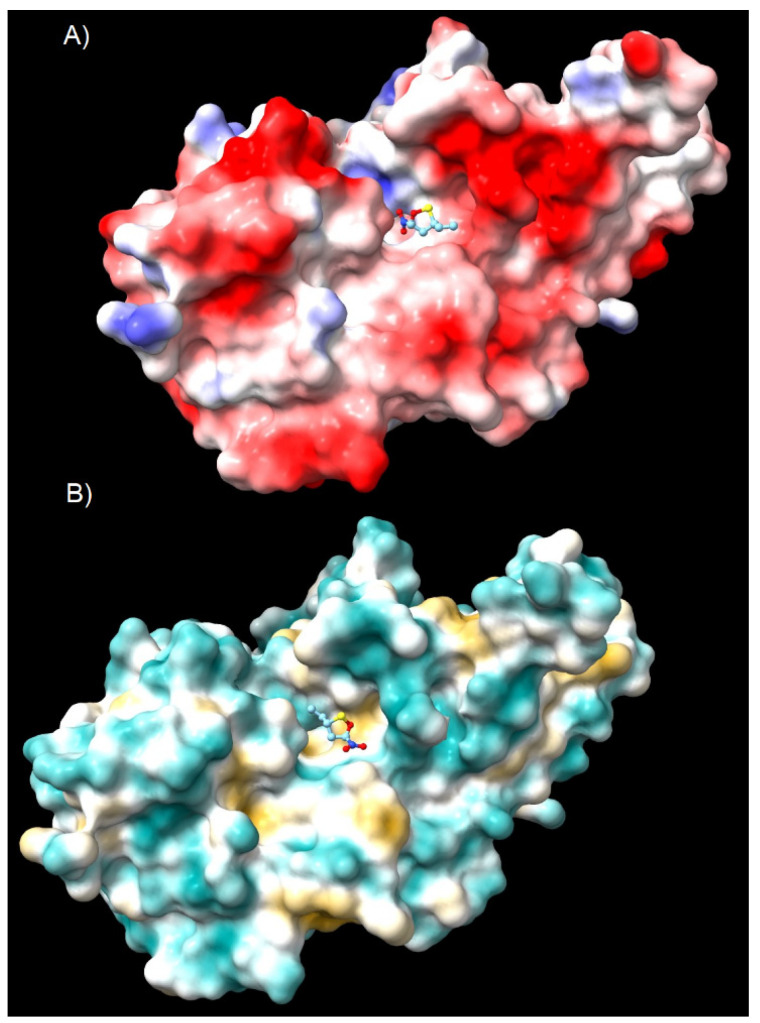
Visualization of (**A**) electrostatic and (**B**) hydrophobic–hydrophilic potential of CYP51 (PDB:1EA1) with **6a** in the pocket.

We also confirmed that the ligand, in all the analyzed cases, was placed in the active pocket (Figure 10), and both could be further characterized by their same size and their hydrophobic–hydrophilic and electrostatic potential.

## 3. Computational Details

The exploration of the reaction profiles was performed on the basis of quantum–chemical calculations with the implementation of the wB97XD/6-31G(d) level of theory using the Gaussian 09 software package [50]. The same function has already been successfully used to explore regio- and stereoselectivity as well as the mechanistic aspects of different types of cycloaddition processes, including [3 + 2] cycloaddition reactions involving different types of TACs [75,76,77]. All the optimized stationary points were characterized using vibrational analysis. It was found that the molecules, intermediates, and products had positive Hessian matrices. On the other hand, all the transition states (**TSs**) showed only one negative eigenvalue in their Hessian matrices. Based on the calculated values of the enthalpies and entropies, the respective Gibbs free energies were calculated using standard equations. The calculations were performed for T = 297 K.

Intrinsic reaction coordinate (IRC) calculations were performed for all the localized transition states. The presence of the solvent in the reaction environment (toluene, nitromethane) was included using the IEFPCM algorithm [78]. The global electron density transfer (GEDT) [79] was calculated according to the following formula:

GEDT = −ΣqA

where qA is the net charge, and the sum is taken over all the atoms of nitroalkene.

The same level of theory was used within the ELF analysis.

The global electronic properties of the reactants were estimated according to the equations recommended earlier by *Parr* and *Domingo* [80,81,82]. According to *Domingo’s* recommendation, the B3LYP/6-31G(d) level of theory was used. All the molecules were fully optimized. Next, the electronic chemical potentials (μ) and chemical hardness (η) were evaluated in terms of one-electron energies of FMO (E_HOMO_ and E_LUMO_) using the following equations:

μ ≈ (E_HOMO_ + E_LUMO_)/2  η ≈ E_LUMO_ − E_HOMO_

Next, the values of μ and η were further used for the calculation of the global electrophilicity (ω) according to the following formula:

ω = μ^2^/2η

Subsequently, global nucleophilicity (N) [83] can be presented as per the following equation:

N = E_HOMO_ − E_HOMO (tetracyanoethene)_

The local electrophilicity (ω*_k_*) corresponding to atom k was calculated by projecting the index ω onto any reaction center k in the molecule using Parr functions, P^+^*_k_* [84]:

ω*_k_* = P^+^*_k_*·ω

The local nucleophilicity (N*_k_*) corresponding to atom *k* was calculated using the global nucleophilicity N and *Parr* functions, P^−^*_k_* [84]_,_ according to the formula:

N*_k_* = P^−^*_k_*·N

The in silico molecular docking was performed using the AutoDock Vina 1.2.0 software implemented by SwissDock [85,86]. It can be said that this is the most widely used tool to predict the binding energy of molecules to target proteins. All the ligand geometries were optimized with the Gaussian16 software [50], and the Merz–Singh–Kollman scheme [87] was used to calculate the partial charges as well. For the docking, the flexible mode was chosen, and for the native ligands, we used the protein files from the RSCB PDB database [88], and all residues were considered within a radius of about 5 Å from the native ligand position.

## 4. Conclusions

Our wB97XD/6-31G(d) (PCM) computational study delivered comprehensive information regarding the regio- and stereoselectivity as well as the molecular mechanism of [3 + 2] cycloaddition reactions with the participation of *syn*-propanethial S-oxide and conjugated nitroalkenes. Independently of the substituent in the 1 position of the nitrovinyl moiety of the nitroalkene, the cycloaddition path leading to 3,4-trans-4-nitro-4-R-1,2-oxathiolanes was always favored from a kinetic point of view. The formation of these types of products is a consequence of local nucleophile/electrophile interactions. From a mechanistic point of view, 32CAs with the participation of nitroethene and its methyl- and chloro-substituted analogs should be considered as a polar but, surprisingly, one-step process. The replacement of the non-polar toluene with more polar solvents (acetone, nitromethane, water) was not sufficient to enforce another type of reaction mechanism. On the other hand, analogous cycloaddition between *syn*-propanethial S-oxide and 1,1-dinitroethene was realized via a stepwise mechanism, because in the reaction path, a zwitterionic intermediate was observed. The evaluation of the binding affinity of the model reaction products to several target proteins has shown that these compounds could exhibit bioactive potential. We confirmed our previous statement that the nitro group can increase the biological activity of these compounds through the creation of hydrogen bonds with proteins.

## Figures and Tables

**Table 1:** Kinetic and thermodynamic parameters of the [3 + 2] cycloaddition between *Syn*-propanethial S-oxide (**1**) and nitroethenes **2a**–**d** in toluene solution according to wB97XD/6-31G(d) (PCM) calculations (for results in other solvents, please see Table S1 in the Supplementary Materials).

Reaction	Transition	∆H	∆S	∆G
**1** + **2a**	**1** + **2a→MCA**	−4.1	−32.7	5.6
	**1** + **2a→TSA**	18.9	−49.2	33.6
	**1** + **2a→3a**	−32.4	−50.3	−17.4
	**1** + **2a→MCB**	−4.3	−38.3	7.1
	**1** + **2a→TSB**	16.0	−47.7	30.2
	**1** + **2a→4a**	−35.6	−48.7	−21.1
	**1** + **2a→MCC**	−3.1	−32.5	6.6
	**1** + **2a→TSC**	19.4	−47.6	33.6
	**1** + **2a→5a**	−39.4	−50.1	−24.4
	**1** + **2a→MCD**	−3.6	−32.6	6.2
	**1** + **2a→TSD**	17.1	−48.4	31.6
	**1** + **2a→6a**	−38.6	−48.6	−24.1
**1** + **2b**	**1** + **2b→MCA**	−4.3	−33.2	5.6
	**1** + **2b→TSA**	20.9	−49.1	35.5
	**1** + **2b→3b**	−31.8	−53.5	−15.8
	**1** + **2b→MCB**	−4.1	−35.7	6.6
	**1** + **2b→TSB**	17.4	−50.3	32.4
	**1** + **2b→4b**	−33.6	−54.1	−17.5
	**1** + **2b→MCC**	−4.1	−28.4	4.3
	**1** + **2b→TC**	18.7	−49.3	33.4
	**1** + **2b→5b**	−40.5	−51.2	−25.2
	**1** + **2b→MCD**	−4.2	−35.2	6.3
	**1** + **2b→TSD**	15.3	−48.8	29.8
	**1** + **2b→6b**	−39.6	−51.0	−24.4
**1** + **2c**	**1** + **2c→MCA**	−4.7	−33.2	5.2
	**1** + **2c→TSA**	16.4	−51.1	31.6
	**1** + **2c→3c**	−32.9	−53.1	−17.1
	**1** + **2c→MCB**	−4.6	−32.3	5.0
	**1** + **2c→TSB**	15.1	−51.6	30.5
	**1** + **2c→4c**	−34.7	−53.0	−18.9
	**1** + **2c→MCC**	−3.8	−33.7	6.3
	**1** + **2c→TC**	17.8	−48.6	32.3
	**1** + **2c→5c**	−41.1	−50.6	−26.1
	**1** + **2c→MCD**	−3.8	−33.9	6.4
	**1** + **2c→TSD**	14.7	−48.9	29.3
	**1** + **2c→6c**	−40.5	−49.9	−25.7
**1** + **2d**	**1** + **2d→MCA**	−5.8	−37.7	5.4
	**1** + **2d→TS1A**	0.9	−49.6	15.7
	**1** + **2d→IA**	−0.1	−49.7	14.7
	**1** + **2d→TS2A**	5.6	−55.4	22.1
	**1** + **2d→3d**	−36.6	−55.2	−20.1
	**1** + **2d→MC**	−4.3	−39.2	7.4
	**1** + **2d→TSC**	12.1	−51.5	27.5
	**1** + **2d→5d**	−44.4	−53.2	−28.6

**Table 2:** Key parameters of critical structures of the [3 + 2] cycloaddition between *Syn*-propanethial S-oxide (**1**) and nitroethenes **2a**–**d** in toluene solution according to wB97XD/6-31G(d) (PCM) calculations (for results in other solvents, please see Table S1 in the Supplementary Materials).

Reaction	Path	Structure	Interatomic Distance [Å]					GEDT [e]
			O1-S2	S2-C3	C3-C4(5)	C4-C5	C5(4)-O1	
**1 + 2a**	**A**	**MCA**	1.498	1.616	4.532	1.322	2.867	0.00
		**TSA**	1.578	1.654	2.704	1.419	1.688	0.45
		**3a**	1.690	1.827	1.555	1.545	1.419	
	**B**	**MCB**	1.499	1.616	3.645	1.322	2.952	0.00
		**TSB**	1.567	1.649	2.552	1.405	1.766	0.33
		**4a**	1.699	1.844	1.539	1.538	1.407	
	**C**	**MCC**	1.497	1.616	4.334	1.320	2.884	0.00
		**TSC**	1.527	1.685	2.070	1.377	2.303	0.20
		**5a**	1.728	1.837	1.536	1.521	1.364	
	**D**	**MCD**	1.498	1.615	4.550	1.319	2.959	0.00
		**TSD**	1.511	1.683	2.014	1.381	2.494	0.24
		**6a**	1.715	1.822	1.540	1.544	1.372	
**1** + **2b**	**A**	**MCA**	1.498	1.616	3.588	1.326	3.144	0.00
		**TSA**	1.576	1.654	2.711	1.421	1.707	0.45
		**3b**	1.694	1.848	1.552	1.544	1.402	
	**B**	**MCB**	1.499	1.615	3.747	1.325	3.108	0.00
		**TSB**	1.578	1.637	2.767	1.431	1.655	0.38
		**4b**	1.706	1.845	1.548	1.545	1.407	
	**C**	**MCC**	1.497	1.616	4.250	1.325	2.993	0.00
		**TSC**	1.526	1.683	2.091	1.381	2.351	0.17
		**5b**	1.720	1.840	1.535	1.525	1.372	
	**D**	**MCD**	1.497	1.616	4.001	1.324	3.012	0.00
		**TSD**	1.512	1.683	2.036	1.383	2.545	0.20
		**6b**	1.708	1.823	1.541	1.546	1.380	
**1** + **2c**	**A**	**MCA**	1.500	1.616	3.811	1.323	2.863	0.00
		**TSA**	1.594	1.650	2.760	1.441	1.587	0.53
		**3c**	1.699	1.850	1.547	1.546	1.398	
	**B**	**MCB**	1.499	1.616	3.885	1.322	2.865	0.00
		**TSB**	1.601	1.642	2.719	1.457	1.645	0.53
		**4c**	1.706	1.847	1.541	1.542	1.401	
	**C**	**MCC**	1.497	1.617	4.185	1.321	2.945	0.00
		**TSC**	1.518	1.685	2.041	1.381	2.413	0.20
		**5c**	1.735	1.831	1.532	1.521	1.348	
	**D**	**MCD**	1.496	1.616	4.061	1.321	2.949	0.00
		**TSD**	1.507	1.681	2.038	1.380	2.575	0.24
		**6c**	1.726	1.823	1.532	1.537	1.354	
**1** + **2d**	**A**	**MCA**	1.503	1.617	3.950	1.321	2.631	0.00
		**TS1A**	1.550	1.620	3.490	1.391	1.813	0.54
		**IA**	1.594	1.617	3.439	1.455	1.524	0.73
		**TS2A**	1.614	1.662	2.523	1.474	1.510	0.61
		**3d**	1.692	1.830	1.544	1.554	1.409	
	**C**	**MC**	1.499	1.616	3.887	1.318	2.775	0.00
		**TS**	1.493	1.677	2.033	1.383	2.641	0.36
		**5d**	1.731	1.823	1.536	1.527	1.348	

**Table 3:** The best docking affinities for the tested oxathiolanes to all tested protein targets.

	CYP51 1EA1	MMP-9 4XCT	COX-1 3KK6	COX-2 5KIR
	**ΔG (kcal/mol)**			
**3a**	−3.79	−5.48	−4.70	−4.43
**4a**	−3.35	−4.95	−4.30	−4.05
**5a**	−3.60	−5.13	−4.55	−4.56
**6a**	−3.49	−4.84	−4.33	−4.25

## Data Availability

Data is contained within the article or Supplementary Materials.

## Supplementary Materials

The following supporting information can be downloaded at: https://www.mdpi.com/article/10.3390/molecules29204892/s1, Table S1: Kinetic and thermodynamic parameters for the [3+2] cycloaddition between *Syn*-propanethial S-oxide (**1**) and nitroethene **2a** according to the wB97XD/6-31G(d) (PCM) calculations; Table S2: Key parameters of critical structures of the [3+2] cycloaddition between *Syn*-propanethial S-oxide (**1**) and nitroethenes **2a**–**d** according to the wB97XD/6-31G(d) (PCM) calculations.
