# Supplementary material for: Syn-Propanethial S-Oxide as an Available Natural Building Block for the Preparation of Nitro-Functionalized, Sulfur-Containing Five-Membered Heterocycles: An MEDT Study
Source: Molecules. 2024 Oct 15;29(20):4892. doi: 10.3390/molecules29204892 (PMC11510298; doi:10.3390/molecules29204892)
Supplement: Supplementary file 1 [file molecules-29-04892-s001.zip › molecules-3224791-supplementary.pdf]

SUPPLEMENTARY MATERIAL

Syn-Propanethial S-Oxide as an Available Natural Building Block for the Preparation of Nitro-Functionalized, Sulfur-Containing Five-Membered Heterocycles: An MEDT Study

Mikołaj Sadowski [1], Ewa Dresler [2], Karolina Zawadzińska [3], Aneta Wróblewska [4] and Radomir Jasiński [1,*]

[1] Department of Organic Chemistry and Technology, Cracow University of Technology, Warszawska 24, 31-155 Krakow, Poland; mikolaj.sadowski@doktorant.pk.edu.pl
[2] Łukasiewicz Research Network—Institute of Heavy Organic Synthesis "Blachownia", Energetyków 9, 47-225 Kędzierzyn-Koźle, Poland; ewa.dresler@icso.lukasiewicz.gov.pl
[3] Radom Scientific Society, Rynek 15, 26-600 Radom, Poland; karolina.zawadzinska@doktorant.pk.edu.pl
[4] Department of Organic Chemistry, University of Lodz, Tamka 12, 91-403 Łódź, Poland; aneta.wroblewska@chemia.uni.lodz.pl
* Correspondence: radomir.jasinski@pk.edu.pl

Table S1. Kinetic and thermodynamic parameters for the [3+2] cycloaddition between *Syn*-propanethial S-oxide (**1**) and nitroethene **2a** according to the wB97XD/6-31G(d) (PCM) calculations.

Reaction	Solvent	Transition	ΔH	ΔS	ΔG
**1+2a**	Toluene	**1+2a→MCA**	-4.1	-32.7	5.6
		**1+2a→TSA**	**18.9**	-49.2	**33.6**
		**1+2a→3a**	-32.4	-50.3	-17.4
		**1+2a→MCB**	-4.3	-38.3	7.1
		**1+2a→TSB**	**16.0**	-47.7	**30.2**
		**1+2a→4a**	-35.6	-48.7	-21.1
		**1+2a→MCC**	-3.1	-32.5	6.6
		**1+2a→TSC**	**19.4**	-47.6	**33.6**
		**1+2a→5a**	-39.4	-50.1	-24.4
		**1+2a→MCD**	-3.6	-32.6	6.2
		**1+2a→TSD**	**17.1**	-48.4	**31.6**
		**1+2a→6a**	-38.6	-48.6	-24.1
	Acetone	**1+2a→MCA**	-2.4	-31.6	7.0
		**1+2a→TSA**	**18.4**	-50.3	33.4
		**1+2a→3a**	-32.4	-50.0	-17.5
		**1+2a→MCB**	-2.9	-38.1	8.4
		**1+2a→TSB**	**15.4**	-50.0	30.3
		**1+2a→4a**	-33.0	-48.9	-18.5

		**1+2a→MCC**	-2.4	-31.1	6.8
		**1+2a→TSAC**	**20.0**	-48.2	34.4
		**1+2a→5a**	-37.5	-49.8	-22.7
		**1+2a→MCD**	-2.5	-34.3	7.7
		**1+2a→TSD**	**16.6**	-47.4	30.7
		**1+2a→6a**	-36.8	-48.6	-22.4
	Nitromethane	**1+2a→MCA**	-2.3	-31.7	7.1
		**1+2a→TSA**	**18.3**	-50.4	33.3
		**1+2a→3a**	-32.3	-49.8	-17.5
		**1+2a→MCB**	-2.3	-31.9	7.2
		**1+2a→TSB**	**15.3**	-50.0	30.2
		**1+2a→4a**	-32.8	-48.9	-18.3
		**1+2a→MCC**	-3.1	-36.4	7.7
		**1+2a→TSAC**	**20.0**	-48.4	34.4
		**1+2a→5a**	-37.4	-49.7	-22.6
		**1+2a→MCD**	-3.1	-37.9	8.2
		**1+2a→TSD**	**16.5**	-47.0	30.5
		**1+2a→6a**	-36.7	-48.6	-22.2
	Water	**1+2a→MCA**	-2.3	-29.7	6.6
		**1+2a→TSA**	**18.2**	-50.4	33.3
		**1+2a→3a**	-32.3	-49.7	-17.4
		**1+2a→MCB**	-2.2	-32.0	7.3
		**1+2a→TSB**	**15.2**	-50.0	30.1
		**1+2a→4a**	-32.7	-48.8	-18.2
		**1+2a→MCC**	-2.8	-34.3	7.4
		**1+2a→TSAC**	**20.0**	-48.6	34.5
		**1+2a→5a**	-37.3	-49.7	-22.5
		**1+2a→MCD**	-2.5	-33.1	7.4
		**1+2a→TSD**	**16.4**	-47.0	30.5
		**1+2a→6a**	-36.6	-48.7	-22.1
**1+2b**	Toluene	**1+2b→MCA**	-4.3	-33.2	5.6
		**1+2b→TSA**	**20.9**	-49.1	**35.5**
		**1+2b→3b**	-31.8	-53.5	-15.8
		**1+2b→MCB**	-4.1	-35.7	6.6
		**1+2b→TSB**	**17.4**	-50.3	**32.4**
		**1+2b→4b**	-33.6	-54.1	-17.5
		**1+2b→MCC**	-4.1	-28.4	4.3
		**1+2b→TC**	**18.7**	-49.3	**33.4**
		**1+2b→5b**	-40.5	-51.2	-25.2
		**1+2b→MCD**	-4.2	-35.2	6.3
		**1+2b→TSD**	**15.3**	-48.8	**29.8**
		**1+2b→6b**	-39.6	-51.0	-24.4
**1+2c**	Toluene	**1+2c→MCA**	-4.7	-33.2	5.2
		**1+2c→TSA**	**16.4**	-51.1	**31.6**
		**1+2c→3c**	-32.9	-53.1	-17.1
		**1+2c→MCB**	-4.6	-32.3	5.0
		**1+2c→TSB**	**15.1**	-51.6	**30.5**
		**1+2c→4c**	-34.7	-53.0	-18.9
		**1+2c→MCC**	-3.8	-33.7	6.3
		**1+2c→TC**	**17.8**	-48.6	**32.3**
		**1+2c→5c**	-41.1	-50.6	-26.1

		**1+2c→MCD**	-3.8	-33.9	6.4
		**1+2c→TSD**	**14.7**	-48.9	**29.3**
		**1+2c→6c**	-40.5	-49.9	-25.7
**1+2d**	Toluene	**1+2d→MCA**	-5.8	-37.7	5.4
		**1+2d→TS1A**	**0.9**	-49.6	15.7
		**1+2d→IA**	-0.1	-49.7	14.7
		**1+2d→TS2A**	**5.6**	-55.4	22.1
		**1+2d→3c**	-36.6	-55.2	-20.1
		**1+2d→MC**	-4.3	-39.2	7.4
		**1+2d→TSC**	**12.1**	-51.5	27.5
		**1+2d→5c**	-44.4	-53.2	-28.6

Table S2. Key parameters of critical structures of the [3+2] cycloaddition between *Syn*-propanethial S-oxide (**1**) and nitroethenes **2a-d** according to the wB97XD/6-31G(d) (PCM) calculations.

Solvent	Reaction	Path	Structure	Interatomic distance [Å]					GEDT [e]
				O1-S2	S2-C3	C3-C4(5)	C4-C5	C5(4)-O1	
Toluene	**1+2a**	**A**	**MCA**	1.498	1.616	4.532	1.322	2.867	0.00
			**TSA**	1.578	1.654	2.704	1.419	1.688	0.45
			**3a**	1.690	1.827	1.555	1.545	1.419	
		**B**	**MCB**	1.499	1.616	3.645	1.322	2.952	0.00
			**TSB**	1.567	1.649	2.552	1.405	1.766	0.33
			**4a**	1.699	1.844	1.539	1.538	1.407	
		**C**	**MCC**	1.497	1.616	4.334	1.320	2.884	0.00
			**TSC**	1.527	1.685	2.070	1.377	2.303	0.20
			**5a**	1.728	1.837	1.536	1.521	1.364	
		**D**	**MCD**	1.498	1.615	4.550	1.319	2.959	0.00
			**TSD**	1.511	1.683	2.014	1.381	2.494	0.24
			**6a**	1.715	1.822	1.540	1.544	1.372	
	**1+2b**	**A**	**MCA**	1.498	1.616	3.588	1.326	3.144	0.00
			**TSA**	1.576	1.654	2.711	1.421	1.707	0.45
			**3b**	1.694	1.848	1.552	1.544	1.402	
		**B**	**MCB**	1.499	1.615	3.747	1.325	3.108	0.00
			**TSB**	1.578	1.637	2.767	1.431	1.655	0.38
			**4b**	1.706	1.845	1.548	1.545	1.407	
		**C**	**MCC**	1.497	1.616	4.250	1.325	2.993	0.00
			**TSC**	1.526	1.683	2.091	1.381	2.351	0.17
			**5b**	1.720	1.840	1.535	1.525	1.372	
		**D**	**MCD**	1.497	1.616	4.001	1.324	3.012	0.00
			**TSD**	1.512	1.683	2.036	1.383	2.545	0.20
			**6b**	1.708	1.823	1.541	1.546	1.380	
	**1+2c**	**A**	**MCA**	1.500	1.616	3.811	1.323	2.863	0.00
			**TSA**	1.594	1.650	2.760	1.441	1.587	0.53
			**3c**	1.699	1.850	1.547	1.546	1.398	
		**B**	**MCB**	1.499	1.616	3.885	1.322	2.865	0.00
			**TSB**	1.601	1.642	2.719	1.457	1.645	0.53
			**4c**	1.706	1.847	1.541	1.542	1.401	
		**C**	**MCC**	1.497	1.617	4.185	1.321	2.945	0.00

			**TSC**	1.518	1.685	2.041	1.381	2.413	0.20
			**5c**	1.735	1.831	1.532	1.521	1.348	
		**D**	**MCD**	1.496	1.616	4.061	1.321	2.949	0.00
			**TSD**	1.507	1.681	2.038	1.380	2.575	0.24
			**6c**	1.726	1.823	1.532	1.537	1.354	
	**1+2d**	A	**MCA**	1.503	1.617	3.950	1.321	2.631	0.00
			**TS1A**	1.550	1.620	3.490	1.391	1.813	0.54
			**IA**	1.594	1.617	3.439	1.455	1.524	0.73
			**TS2A**	1.614	1.662	2.523	1.474	1.510	0.61
			**3d**	1.692	1.830	1.544	1.554	1.409	
		C	**MC**	1.499	1.616	3.887	1.318	2.775	0.00
			**TS**	1.493	1.677	2.033	1.383	2.641	0.36
			**5d**	1.731	1.823	1.536	1.527	1.348	
Acetone	**1+2a**	A	**MCA**	1.504	1.615	4.343	1.322	2.968	0.00
			**TSA**	1.604	1.656	2.717	1.450	1.561	0.58
			**3a**	1.701	1.848	1.536	1.531	1.411	
		B	**MCB**	1.504	1.615	3.637	1.322	3.057	0.00
			**TSB**	1.595	1.641	2.674	1.446	1.574	0.55
			**4a**	1.698	1.842	1.543	1.540	1.410	
		C	**MCC**	1.504	1.615	4.011	1.321	2.981	0.00
			**TSC**	1.526	1.686	2.028	1.380	2.363	0.23
			**5a**	1.730	1.838	1.536	1.521	1.365	
		D	**MCD**	1.504	1.614	4.544	1.320	3.144	0.00
			**TSD**	1.506	1.682	1.985	1.383	2.657	0.29
			**6a**	1.718	1.822	1.539	1.543	1.373	
Nitromethane	**1+2a**	A	**MCA**	1.504	1.614	4.324	1.322	2.981	0.00
			**TSA**	1.606	1.656	2.709	1.452	1.556	0.59
			**3a**	1.701	1.848	1.536	1.530	1.411	
		B	**MCB**	1.504	1.615	3.636	1.322	3.069	0.00
			**TSB**	1.597	1.641	2.667	1.447	1.569	0.56
			**4a**	1.698	1.842	1.543	1.540	1.410	
		C	**MCC**	1.504	1.615	3.982	1.321	2.990	0.00
			**TSC**	1.526	1.686	2.024	1.381	2.368	0.23
			**5a**	1.730	1.838	1.536	1.521	1.365	
		D	**MCD**	1.504	1.614	4.546	1.320	3.163	0.00
			**TSD**	1.506	1.682	1.984	1.383	2.669	0.29
			**6a**	1.718	1.822	1.539	1.543	1.373	
Water	**1+2a**	A	**MCA**	1.505	1.614	4.304	1.322	2.998	0.00
			**TSA**	1.607	1.657	2.703	1.453	1.552	0.59
			**3a**	1.701	1.848	1.536	1.530	1.411	
		B	**MCB**	1.505	1.615	3.637	1.322	3.074	0.00
			**TSB**	1.598	1.642	2.662	1.448	1.566	0.56
			**4a**	1.698	1.842	1.544	1.540	1.410	
		C	**MCC**	1.505	1.615	3.820	1.321	2.991	0.00
			**TSC**	1.526	1.686	2.022	1.381	2.371	0.24
			**5a**	1.730	1.838	1.536	1.521	1.365	
		D	**MCD**	1.505	1.614	4.547	1.320	3.183	0.00
			**TSD**	1.505	1.682	1.979	1.384	2.715	0.30
			**6a**	1.719	1.822	1.539	1.543	1.373	
